# Successful Magnetic Resonance Imaging-Guided Focused Ultrasound Surgery for Recurrent Uterine Fibroid Previously Treated with Uterine Artery Embolization 

**DOI:** 10.1155/2010/351273

**Published:** 2010-08-16

**Authors:** Sang-Wook Yoon, Kyoung Ah Kim, Sang Heum Kim, Jong Tae Lee

**Affiliations:** Department of Diagnostic Radiology, CHA Bundang Medical Center, CHA University, 351 Yatap-dong, Bundang-gu, Sungnam-si, Gyunggi-do 463-712, Republic of Korea

## Abstract

A 45-year-old premenopausal woman was referred to our clinic due to recurring symptoms of uterine fibroids, nine years after a uterine artery embolization (UAE). At the time of screening, the patient presented with bilateral impairment and narrowing of the uterine arteries, which increased the risk of arterial perforation during repeated UAE procedures. The patient was subsequently referred for magnetic resonance imaging-guided focused ultrasound surgery (MRgFUS) treatment. Following the treatment, the patient experienced a significant improvement in symptoms (symptom severity score was reduced from 47 to 12 by 1 year post-treatment). MR images at 3 months showed a 49% decrease in fibroid volume. There were no adverse events during the treatment or the follow-up period. This case suggests that MRgFUS can be an effective treatment option for patients with recurrent fibroids following previous UAE treatment.

## 1. Introduction

Uterine leiomyoma (fibroid) is the most common reproductive tract tumor in women of reproductive age. Fibroids have been clinically identified in at least 25% of women [1], and pathological analysis suggests that the prevalence of fibroids may be as high as 77% [2]. Symptomatic fibroids can significantly affect quality of life (QOL) and can result in heavy and prolonged menstrual flow, urinary frequency, pelvic pain, abdominal pressure, infertility, and dyspareunia [3–5]. 

Surgical treatments for uterine fibroids include hysterectomy and myomectomy [6]. Minimally invasive or noninvasive treatments include uterine artery embolization (UAE), magnetic resonance imaging-guided focused ultrasound surgery (MRgFUS), and hormonal therapy [6–9]. Each of these treatment options, which require minimal or no hospitalization, enables women to preserve their uteri [10] and usually minimize complications, recovery time, and treatment costs [11, 12].

UAE is a minimally invasive, image-guided therapy, in which the blood supply to the uterine fibroid is blocked by catheterization, and the ischemic necrosis of the fibroids is induced by the insertion of embolic particles [13]. The embolic particles are usually composed of polyvinyl alcohol, tris-acryl, or gelatin sponge material. 

MRgFUS is a noninvasive treatment in which ultrasound energy, focused on the fibroid in multiple focal spots, raises the temperature of tissue within the focal zone and causes coagulative necrosis. MRI guides and monitors the procedure, thereby providing closed loop anatomical and thermal feedback [9]. 

Several measures are used to assess the efficacy of these minimally invasive or noninvasive treatments, including a Uterine Fibroids Symptoms Quality Of Life (UFS-QOL) assessment questionnaire [14], fibroid shrinkage, and patient satisfaction. As with any fibroid treatment, besides hysterectomy, symptoms can recur following the less invasive approaches. Consequently, referral to an alternative treatment, after a particular modality has been pursued, is also a measure of the treatment efficacy.

Different patient selection criteria are established for UAE and MRgFUS treatments. For UAE, submucosal and pedunculated fibroids may be considered as relative contraindications, as is a previous internal iliac or uterine artery occlusion, or a recent GnRH analogue administration. In addition, there is insufficient data to advocate UAE as a means of preserving fertility [15, 16]. For MRgFUS, hyper-intense fibroids and multiple fibroids may be considered relative contraindications, as they are difficult to treat. In addition, in cases where the ultrasound beam is interrupted by anatomical structures, such as bowels, bones, or nerves, MRgFUS treatment may be impossible without successful mitigation techniques [17].

This is the first case report of MRgFUS treatment in a patient with recurring fibroid symptoms following UAE.

## 2. Case Report

**Figure 1:**
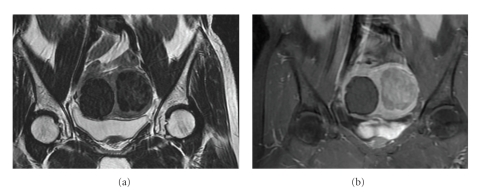
Screening MR images: (a) coronal T2-weighted image showing two fibroids, (b) coronal T1-weighted contrast-enhanced image—the right fibroid is already nonenhancing, whereas the left one is still viable.

**Figure 2:**
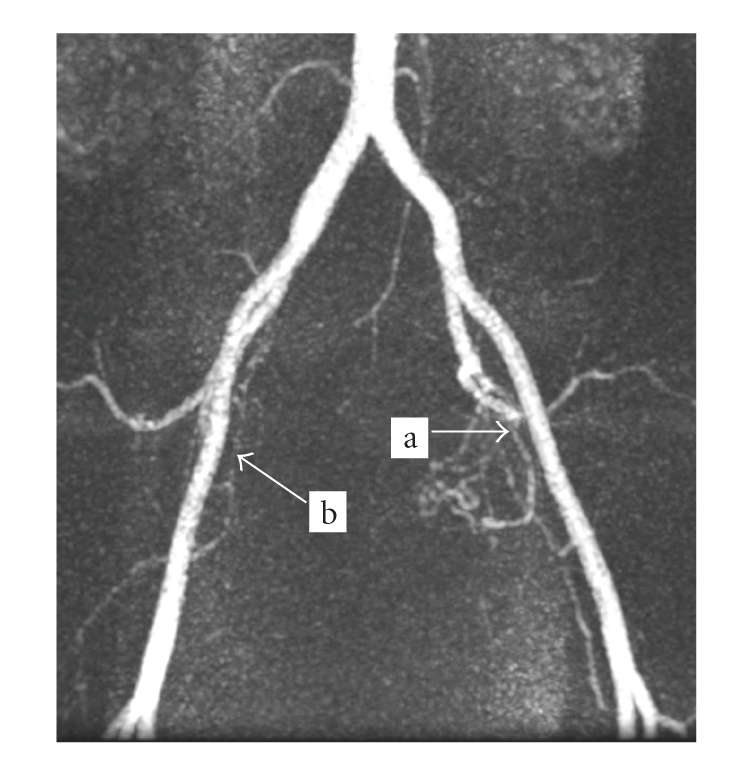
MR angiography. The reason for UAE unsuitability: (a) narrow left uterine artery, (b) no obvious right uterine artery.

A 45-year-old premenopausal woman, with a BMI of 22.1 and 2 previous pregnancies, complained of menorrhagia in 1998. Clinical examination showed two intramural fibroids with volumes of approximately 115 cc and 15 cc. In November 1998, the patient underwent a UAE, and both her fibroids were treated. Approximately nine years later, in 2008, the patient reported the recurrence of symptoms, including severe menorrhagia and irregular menstrual periods (symptom severity score of 47). A pelvic MRI including MR angiography was performed in order to determine her fibroid status and suitability for an additional UAE. Two intramural fibroids were observed (Figure 1). The first was an 81 cc fibroid on the right side of the uterus, which was nonenhancing on contrast-enhanced T1-weighted images (probably due to necrosis following the previous UAE procedure). The second fibroid, which was located on the left side of the uterus, was 90 cc and was enhancing on T1-weighted images. An MR angiography revealed that the right fibroid lacked a blood supply, with an almost invisible right uterine artery. The left fibroid was supplied only by the narrow left uterine artery (Figure 2). The left fibroid was likely a recurring or new fibroid that had not been treated by the previous UAE. It was recommended that the patient not undergo an additional UAE, due to the difficulty in approaching the fibroid bilaterally and the increased risk of arterial perforation during repeated UAE procedures. Since the patient insisted on a noninvasive treatment for her symptoms, she was referred to our unit for MRgFUS treatment. 

Following a negative endometrial biopsy result, the left fibroid was deemed suitable for MRgFUS treatment. 

The MRgFUS procedure was performed using the Exablate 2000 system (InSightec Ltd., Haifa, Israel) and the 1.5T HDx MRI (GE Healthcare, Milwaukee, U.S.). Patient preparation included shaving and cleaning of the abdomen, insertion of a urinary catheter, and administration of conscious sedation (Fentanyl, one ampoule). The patient was then placed on the ExAblate treatment table with her abdomen positioned over the ultrasound transducer bath. 

**Figure 3:**
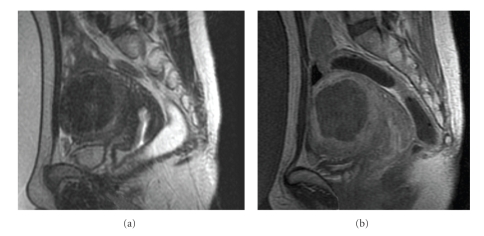
Treatment images: (a) Sagittal T2-weighted planning image, (b) Sagittal contrast-enhanced T1-weighted posttreatment image showing 90% of nonenhancing volume on the left fibroid.

Pretreatment T2-weighted MR images were obtained for procedure planning and for targeting the left fibroid. For the duration of the treatment, 35 sonications were delivered over approximately 1 hour, and thermal responses consistent with effective ablation were observed on the real-time temperature maps. T1-weighted contrast-enhanced images that were obtained immediately following the procedure showed a nonperfused volume (NPV) of 81 cc, which constitutes approximately 90% of the fibroid volume (Figure 3).

The patient was discharged approximately 30 minutes after completion of the procedure and reported a return to normal activity and a regular work schedule after one day. The patient did not report any pain and was very satisfied with her rapid recovery compared to her previous UAE. There were no adverse events during or after the treatment.

**Figure 4:**
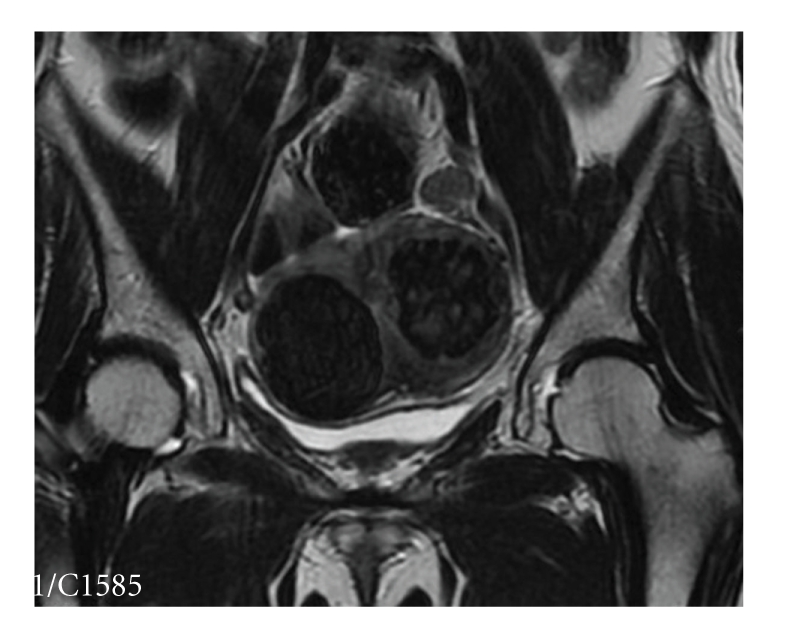
Coronal T2-weighted image three months post-treatment, showing 49% volume shrinkage of the treated fibroid.

Three months after the treatment, the patient reported significant symptom improvement. Contrast-enhanced T1-weighted and T2-weighted MR images, obtained at that time, revealed shrinkage of the treated fibroid by 49% (Figure 4). The patient's SSS was 22, reflecting a 25-point decrease from the base-line score before the MRgFUS treatment. At the one-year follow-up assessment, her symptom severity score was further decreased to 12.

## 3. Discussion

We are currently noticing an increase in the number of uterine fibroid patients who seek minimally invasive or non-invasive treatment options. These options include laparoscopic surgeries, UAE, MRgFUS, and other modalities. 

Patients should be made aware of all the treatment options available for uterine fibroids, including invasive, minimally invasive or noninvasive procedures. The most clinically suitable treatment option should be recommended for each individual patient, according to her medical condition and personal needs. 

UAE treatment may pose an increased risk in cases where the uterine artery is absent in the area of the fibroid, or when a highly tortuous uterine artery or ectopic arterial branches feed the fibroid [18]. Therefore, patients who present with one of these anatomical features, who have recurring symptoms and are seeking a minimally invasive or noninvasive treatment, may be referred for MRgFUS or hormonal therapy.

This paper demonstrates how patients can potentially benefit from alternative minimally invasive or noninvasive treatment options for symptomatic uterine fibroids. Specifically, MRgFUS treatment can be a good option for patients who were previously treated with UAE. Additional studies of the safety and efficacy of MRgFUS following UAE should be conducted.
